# Efficacy comparison of two doses of dezocine on preventing sufentanil-induced cough in patients undergoing coronary artery bypass grafting surgery: A prospective, randomized controlled trial

**DOI:** 10.1097/MD.0000000000041416

**Published:** 2025-02-07

**Authors:** Chun-mei Xie, Li-xian He, Meng-qi Shen, Yun-tai Yao

**Affiliations:** a Department of Anesthesiology, Fuwai Yunnan Hospital, Chinese Academy of Medical Sciences, Affiliated Cardiovascular Hospital of Kunming Medical University, Kunming, China; b Department of Anesthesiology, Fuwai Hospital, National Center for Cardiovascular Diseases, Peking Union Medical College and Chinese Academy of Medical Sciences, Beijing, China.

**Keywords:** cardiovascular surgery, cough, dezocine, general anesthesia, induction, sufentanil

## Abstract

**Background:**

Sufentanil-induced cough (SIC) is a common but irritating phenomenon during general anesthesia (GA) induction; studies have reported that high doses of dezocine can effectively prevent it. The aim is to explore the efficacy and safety of low-dose dezocine in preventing SIC during GA induction in coronary artery bypass grafting (CABG) surgery.

**Methods:**

81 elective CABG surgery patients were randomly and equally divided into 2 dezocine groups of different doses and 1 control group. Before GA induction, the patients received “pre-injection” solution: 0.1 mg/kg dezocine in the high-dose (HD) group, 0.05 mg/kg dezocine in the low-dose (LD) group or an equal volume of saline in the control (C) group. The primary outcome was the incidence of SIC within 1 minute after sufentanil administration. The secondary outcomes included the severity of SIC, the adverse reactions within 1 minute after injection of the “pre-injection” solution, and the vital signs at various time points.

**Results:**

One patient had moderate SIC in the HD group (3.7%), 3 patients (11.1%) had SIC (1 mild and 2 severe) in the LD group, and 8 patients (29.6%) had SIC (3 mild, 1 moderate, and 4 severe) in the C group. The difference between the HD and the C groups was statistically significant (*P* = .01). In contrast, in comparing the LD and the C groups, the LD and the HD groups had no statistically significant difference (*P* > .017).

**Conclusion:**

The current study suggested that pretreatment of 0.05 mg/kg dezocine neither prevented SIC occurrence nor attenuated SIC severity during GA induction in CABG surgery, but 0.1 mg/kg dezocine did.

## 1. Introduction

Opioids like fentanyl and its derivatives (sufentanil, remifentanil, and alfentanil) are regularly used as general anesthesia (GA) induction drugs because of their strong analgesic properties, short duration of action, and good cardiovascular stability.^[1,2]^ Opioid-induced cough (OIC) is a common but irritating phenomenon during GA induction, which is mostly transient, benign and self-limiting but could be associated with adverse effects such as hypertension, tachycardia, increased intracranial, ocular and abdominal pressures and airway obstruction, with an incidence between 7% and 70% reported in previous studies.^[3–6]^ A lot of pharmacological interventions are used to relieve OIC; the appropriate drugs include lidocaine, dexmedetomidine, dezocine, low-dose fentanyl and magnesium sulfate.^[2–6]^ Non-pharmacological interventions include pre-low dose administration, post-dilution administration, slow administration, peripheral or central vascular administration, huffing maneuver and acupressure or swallowing action before the opioid injection.^[3,7–9]^ The pathogenesis of OIC is complex and remains poorly understood. It may be related to pulmonary chemoreflex, inhibition of the central sympathetic system leading to vagal predominance, reflex bronchoconstriction after the stimulation of tracheobronchial tree receptors, C-fiber receptors, rapid adapting pulmonary stretch receptors, histamine release, opioid receptor dualism and muscle rigidity.^[6,7,10,11]^

Dezocine is an analgesic, acting as a partial μ-receptor agonist and a κ-receptor antagonist.^[12]^ Previous studies have reported that dezocine can effectively prevent OIC.^[2,5,6,13]^ Sufentanil is a powerful opioid analgesic that mainly activates μ-opioid receptors and may cause sufentanil-induced cough (SIC) during GA induction. Moreover, the dosage of sufentanil during GA induction in cardiovascular surgery is significantly higher than in other types of surgery, and the adverse effects caused by SIC, such as hypertension and tachycardia, may be fatal for cardiovascular disease, especially in patients with aortic dissection.^[14]^ Our previous study showed that a 0.1 mg/kg dezocine preinjection could prevent SIC in cardiovascular surgery.^[15]^ However, 20% of adverse reactions were also higher.^[15]^ In addition, dezocine was reported to inhibit fentanyl-induced cough in a dose-dependent manner.^[16]^ Ma et al^[13]^ reported that 0.03 mg/kg dezocine could reduce remifentanil-induced cough during general anesthesia induction. Therefore, we hypothesized that low-dose dezocine (0.05 mg/kg) could be effective in reducing SIC and the adverse reactions could be lower; we designed a prospective, double-blind, randomized controlled trial to explore the efficacy and safety of low-dose (0.05 mg/kg) dezocine in preventing SIC during GA induction in coronary artery bypass grafting (CABG) surgery.

## 2. Material and methods

### 2.1. Study design

The study involved 81 patients scheduled for CABG surgery between May 2022 and October 2022 at Fuwai Yunnan Hospital with the approval of the Local Ethics and Research Committee (2022-038-01). After informed consent, all enrolled patients were randomly and equally divided into the control (C), low-dose (LD) and high-dose (HD) groups using a computer program to generate a random sequence. Inclusion criteria: age ≥ 18 years old; elective CABG surgery for endotracheal intubation under GA. Exclusion criteria: known allergy to dezocine; emergency surgery; abnormal liver function, renal insufficiency (serum creatinine > 42.0 mmol/L); elevated intraocular pressure such as glaucoma; elevated intracranial pressure; chronic pharyngitis, chronic bronchitis, bronchial asthma, chronic obstructive pulmonary disease; upper respiratory tract infection in the last 2 weeks; a long history of opioid use; a history of chronic cough.

### 2.2. Intervention

Patients were randomly allocated into 3 groups using computer-generated codes. Allocation concealment was established by placing the randomization sequence in consecutively numbered, opaque envelopes. The pre-injected solution was prepared and distributed by the anesthetic assistant according to the group and labeled “pre-injection.” Pre-injection solution total 10 mL: HD group extracted 10 mg dezocine (China Yangzijiang Pharmaceutical Group Co., Ltd, China) diluted to 10 mL and prepared into 1 mg/mL. In the LD group, 5 mg dezocine was extracted, diluted to 10 mL, and 0.5 mg/mL was obtained. Group C prepared 10 mL of normal saline.

### 2.3. Anesthesia and research procedure

After entering the operating room, 5-lead electrocardiogram monitoring, pulse oxygen saturation (SpO_2_) and bispectral index (BIS) were connected. After routine disinfection, open venous access was placed in the forearm of the right upper limb, 16 G venous trocar was placed in each of them, and sodium lactate ringer injection was 4–6 mL/min. At the same time, left radial artery puncture catheterization was performed to monitor the invasive arterial blood pressure.

Before GA induction, the patient was asked to cough and phlegm to clear respiratory secretions, adjust to deep and slow breathing, and mask oxygen inhalation 5 L/min. No other drugs were administered to the patients in the operating room before dezocine. First, 0.1 mL/kg “pre-injection” solution was administered intravenously. Immediately after injection, an anesthetic registrar blinded to the group recorded the adverse reactions. One min later, 2 μg/kg sufentanil was administered within 3 seconds (200 μg sufentanil diluted to 20 mL; Yichang Humanwell Pharmaceutical Co., China). Immediately after injection, an anesthetic registrar blinded to the group recorded the incidence of cough as “yes” or “no,” the number of coughs. Depending on the number of coughs observed, the cough severity was graded as mild (1–2), moderate (3–5) and severe (>5). In addition, vital signs at various time points were recorded.

After 1 minute, midazolam 0.1 mg/kg, etomidate 0.2 mg/kg and cisatracurium 0.15 mg/kg (Jiangsu Enhua Pharmaceutical Co., China) were injected successively. Endotracheal intubation was then conducted, and the patients were ventilated mechanically. All patients were given maintenance anesthesia according to anesthesiologist practice.

### 2.4. Observation indicators

The primary outcome was the incidence of SIC within 1 minute of sufentanil administration. The secondary outcomes were the severity of SIC within 1 minute of sufentanil administration, the adverse reactions such as dizziness and drowsiness, nausea, vomiting, cold feeling, truncal rigidity, respiratory depression and others within 1 minute after injection of “pre-injection” solution, and the vital signs at various times points during GA induction. Truncal rigidity: the tension of trunk muscles causes difficulty in mask ventilation and respiratory depression: apnea ≥ 15 seconds. Systolic blood pressure, diastolic blood pressure, mean arterial pressure, heart rate (HR), SpO_2_ and BIS were recorded before “pre-injection” solution injection (T0), 1 minute after “pre-injection” solution injection (T1), 1 minute after sufentanil injection (T2), before endotracheal intubation (T3), and 1 minute after endotracheal intubation (T4). General information such as age, height, weight, American Society of Anesthesiologists physical status classification, type of surgery, specific comorbidities, and smoking history were recorded.

### 2.5. Sample size calculation

In our previous study, the incidence of SIC (2 μg/kg) was about 40% in group C and 0% in group HD.^[15]^ We conducted a preliminary experiment and found that with 0.05 mg/kg dezocine pretreatment, the incidence of SIC was 8%. Therefore, this study required at least 72 patients per group to attain appropriate power (α = 0.05 [2-tailed]; β = 0.2). Considering the dropout, the sample size was increased to 81 patients (27 patients per group).

### 2.6. Statistical analysis

All analyses were conducted using SPSS 24.0 (SPSS Inc., Chicago). Data were tested for normality using the Kolmogorov-Smirnov test and were expressed as numbers and percentages for qualitative variables and a mean ± standard deviations for quantitative ones. Qualitative variables were compared between the groups using the Pearson chi-square test or nonparametric rank sum test (2 samples/multi-samples). Quantitative variables were compared between the groups using a 1-way analysis of variance (ANOVA) followed by a Student–Newman–Keuls post hoc test. The *P* value of <.05 was considered statistically significant. If a significant effect was indicated among 3 groups in the chi-square or Nonparametric rank sum test, pairwise comparisons were followed with a more conservative alpha level of 0.017.

## 3. Results

### 3.1. General clinical data

**Figure 1.**
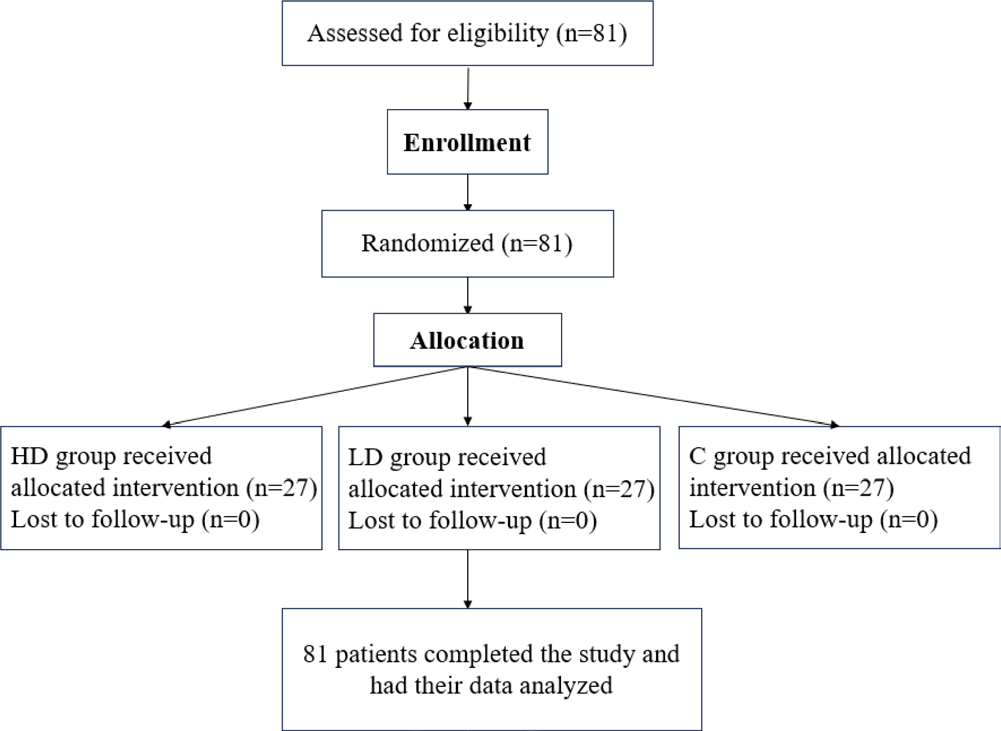
The flowchart shows details of clinical procedures throughout the study.

As shown in Figure 1, 81 patients in the HD (n = 27), LD (n = 27), and C (n = 27) groups completed the study protocol and had their data analyzed. As shown in Table 1, a pairwise comparison of general data such as gender composition, age, height, weight, smoking history, occupational dust exposure and history of taking angiotensin-converting enzyme inhibitors (ACEI) among the 3 groups showed no statistical significance (*P* > .05).

**Table 1:** The general clinical data.

Characteristics	Total	HD (n = 27)	LD (n = 27)	C (n = 27)	*P* value
Age (yr)	61.3 ± 8.3	62.8 ± 6.2	59.7 ± 8.9	61.3 ± 9.3	.39
Height (cm)	169.1 ± 6.9	168.7 ± 6.5	169.5 ± 6.8	169.0 ± 7.5	.91
Weight (kg)	75.8 ± 11.6	77.1 ± 10.3	78.4 ± 13.1	71.7 ± 10.4	.08
Male/female (n)	70/11	24/3	24/3	22/5	.66
ASA III/IV (n)	71/10	23/4	23/4	23/4	.89
Smoking: yes/no (n)	51/30	16/11	20/7	15/12	.33
Occupational dust exposure: yes/no (n)	9/72	1/26	5/22	3/24	.22
ACEI history: yes/no (n)	11/70	3/24	3/24	5/22	.45

Values are expressed as mean ± SD or numbers. One-way ANOVA test was used for data analysis, and none of the variables were significantly different.

ACEI = angiotensin-converting enzyme inhibitors, ASA = American Society of Anesthesiologists, C = control, HD = high-dose dezocine, LD = low-dose dezocine, SD = standard deviation.

### 3.2. SIC incidence

As shown in Table 2, 1 patient (3.7%) had a moderate SIC in the HD group, 3 patients (11.1%) had SIC (1 mild and 2 severe) in the LD group, and 8 patients (29.6%) had SIC (3 mild, 1 moderate, and 4 severe) in the C group. The difference between the HD and C groups was statistically significant (*P* = .01). In contrast, when comparing the LD and C groups, the LD and HD groups had no statistically significant difference (*P* > .017).

**Table 2:** SIC incidence [n (%)].

Outcomes	Group				Pairwise comparisons		
	HD (n = 27)	LD (n = 27)	C (n = 27)	Overall *P* value	HD vs C	HD vs LD	LD vs C
SIC occurrence	1 (3.7%)	3 (11.1%)	8 (29.6%)	.02*	0.01*	0.3	0.09
SIC severity	–	–	–	.02*	0.01*	0.29	0.1
Mild	0	1 (3.7%)	3 (11.1%)	–	–	–	–
Moderate	1 (3.7%)	0	1 (3.7%)	–	–	–	–
Severe	0	2 (7.4%)	4 (14.8%)	–	–	–	–

C = control group, HD = high-dose dezocine group, LD = low-dose dezocine group, SIC = sufentanil-induced cough.

* Indicates that *P* is statistically significant.

### 3.3. Vital signs

As shown in Table 3, BIS at 1 minute after “pre-injection” solution injection (T1), the difference between the HD and C groups, the HD and LD groups was statistically significant (*P* = .01); HR at 1 minute after sufentanil injection (T2), the difference between the HD and C groups was statistically significant (*P* = .01). There were no significant differences in other vital signs among the 3 groups at each time point (*P* > .05; a 1-way ANOVA test was used for data analysis).

**Table 3:** Vital signs at different time points (mean ± SD).

Vital signs	Groups	T0	T1	T2	T3	T4
SBP (mm Hg)	HD (n = 27)	156.8 ± 19.9	161.9 ± 21.9	159.3 ± 25.1	108.9 ± 23.9	118.8 ± 25.2
	LD (n = 27)	152.1 ± 22.0	157.3 ± 24.5	154.8 ± 26.0	104.1 ± 19.4	109.7 ± 17.7
	C (n = 27)	152.1 ± 23.1	152.8 ± 22.5	157.3 ± 21.3	100.6 ± 18.9	105.2 ± 18.3
DBP (mm Hg)	HD (n = 27)	72.9 ± 9.8	74.8 ± 10.3	72.6 ± 11.7	50.3 ± 7.9	59.1 ± 14.0
	LD (n = 27)	74.4 ± 9.8	77.8 ± 10.3	75.7 ± 11.0	53.9 ± 8.1	57.4 ± 9.3
	C (n = 27)	71.7 ± 9.4	72.1 ± 10.3	74.0 ± 10.2	50.3 ± 13.5	54.1 ± 7.9
MAP (mm Hg)	HD (n = 27)	104.7 ± 13.6	107.8 ± 14.9	104.1 ± 16.9	72.8 ± 15.2	78.2 ± 18.2
	LD (n = 27)	104.2 ± 12.5	107.4 ± 14.1	104.9 ± 15.4	71.1 ± 11.2	75.4 ± 11.6
	C (n = 27)	100.1 ± 12.4	101.8 ± 12.9	104.8 ± 11.2	68.5 ± 9.5	71.4 ± 10.4
HR (bpm)	HD (n = 27)	65.8 ± 11.3	61.6 ± 8.3	58.9 ± 11.5†	58.0 ± 11.9	63.3 ± 13.5
	LD (n = 27)	68.3 ± 10.1	65.1 ± 10.6	64.8 ± 11.8†	57.4 ± 8.0	63.6 ± 8.7
	C (n = 27)	67.0 ± 8.5	67.1 ± 8.3	67.2 ± 9.8†	56.5 ± 10.4	60.1 ± 9.5
SpO_2_ (%)	HD (n = 27)	97.9 ± 1.6	99.7 ± 0.8	99.4 ± 2.1	99.7 ± 1.3	99.7 ± 1.3
	LD (n = 27)	97.7 ± 1.8	99.5 ± 0.8	99.8 ± 0.7	99.8 ± 0.6	99.8 ± 0.8
	C (n = 27)	98.4 ± 1.6	99.6 ± 0.9	99.9 ± 0.3	99.7 ± 0.7	99.7 ± 0.7
BIS	HD (n = 27)	94.3 ± 3.3	91.4 ± 4.6*	81.8 ± 15.1	34.4 ± 7.1	34.0 ± 6.4
	LD (n = 27)	96.2 ± 2.8	94.4 ± 3.4*	82.4 ± 14.3	34.1 ± 6.6	34.4 ± 5.7
	C (n = 27)	95.7 ± 2.7	94.2 ± 3.9*	82.3 ± 19.1	34.2 ± 8.5	32.9 ± 4.9

One-way ANOVA test was used for data analysis, none of the variables were significantly different.

BIS = bispectral index, C = control group, DBP = diastolic blood pressure, HD = high-dose dezocine group, HR = heart rate, LD = low-dose dezocine group, MAP = mean arterial pressure, SBP = systolic blood pressure, SD = standard deviation, SpO_2_ = pulse oxygen saturation.

* Indicates the difference between the HD and the C group, the HD and the LD group were statistically significant (*P* = .01).

† Indicates the difference between the HD and the C group was statistically significant (*P* = .01).

### 3.4. Adverse reaction

None of the patients had a cough within 1 minute after intravenous pre-injection solution. As shown in Table 4, the incidence of dizziness and drowsiness was 5 patients in the HD group, 2 in the LD group, and 0 in the C group. There was a statistical difference between the HD and C groups (*P *= .011). In contrast, in comparing the LD and C groups, the LD and HD groups had no statistically significant difference (*P* > .017).

**Table 4:** Occurrence of adverse reactions [n (%)].

Adverse reactions	HD (n = 27)	LD (n = 27)	C (n = 27)	*P* value	Pairwise comparisons		
					HD vs C	HD vs LD	LD vs C
Dizziness and drowsiness	5 (18.5%)	2 (7.4%)	0	.02*	0.01*	0.29	0.10
Nausea	1 (3.7%)	0	0	.37			

C = control group, HD = high-dose dezocine group, LD = low-dose dezocine group.

* Indicates that *P* is statistically significant.

## 4. Discussion

In the current study, the authors found that preinjection of 0.05 mg/kg dezocine did not reduce cough induced by 2 μg/kg sufentanil during anesthesia induction in CABG surgery, but 0.1 mg/kg dezocine did. Also, there were no patients who developed coughs after preinjection of dezocine. Moreover, there was no statistical difference in adverse reactions such as dizziness and drowsiness caused by preinjection of 0.05 mg/kg and 0.1 mg/kg dezocine. In addition, there was no statistical difference in vital signs and anesthesia depth among the groups at most various time points after the preinjection of dezocine. These results were consistent with the meta-analysis, which reported that 0.1 mg/kg dezocine significantly reduced the incidence and severity of SIC in anesthesia induction but had no significant effect on vital signs, and the dezocine-treated patients had a higher occurrence rate of dizziness as compared with placebo.^[6,17]^

The pathogenesis of OIC was complex and remains understood. It might be related to pulmonary chemoreflex, inhibition of the central sympathetic system leading to vagal predominance, reflex bronchoconstriction after the stimulation of tracheobronchial tree receptors, C-fiber receptors, rapid adapting pulmonary stretch receptors, histamine release, opioid receptor dualism and muscle rigidity.^[6,7,10,11]^ Additionally, many factors could contribute to the occurrence of OIC, which could be divided into 2 categories. One was the patients’ physical conditions (age, sex, smoking status, disease history, etc). Another was the usage of opioids (drug category, dosage, concentration, injection site, injection concentration, injection rate, etc).^[4,6,18]^ As the incidence of OIC was high in infants and children even in small doses (1 µg/kg),^[19,20]^ OIC might be more evident in the Asian population than in the European one.^[21]^ Whether smoking increased OIC was controversial. Bailey^[10]^ reported that OIC was noticeable in smokers, and another study reported the incidence of cough in the nonsmoker and smoker group (>10 cigarettes per day) was similar.^[22]^ In the current study, the authors found no statistical differences in age, gender, smoking status, ACEI drug-taking history and occupational dust exposure among the groups. However, due to the limited sample size, there was no statistical analysis on whether the above factors affected the incidence of cough.

Dezocine was a mixed κ and μ opioid agonist-antagonist.^[12]^ Although no longer produced in Western countries, dezocine has been used in China for decades.^[1,2,4–6,13,15–17,23–25]^ The mechanism of dezocine-inhibited SIC was not very clear. Researchers believed that it was possible that dezocine competitively binds to the µ receptor, resulting in the decrease of fentanyl binding to the µ receptor or inhibition of histamine release, thereby alleviating cough response.^[26]^ Another possible explanation was that dezocine suppressed SIC by opioid receptor dualism,^[21]^ κ-receptor antagonism or inhibited norepinephrine and serotonin reuptake, which antagonizes sufentanil-activated μ receptors, reducing cough.^[10,12]^

In terms of the safety of dezocine, the authors found no cough after an intravenous of 0.1/0.05 mg/kg dezocine in the current study, which may show that μ-opioid receptor-mediated cough may be significantly reduced in partial agonism when compared to sufentanil with full agonism or by κ-receptor antagonism. And because of its partial μ agonism, dezocine exhibited a ceiling effect for common opioids-related adverse effects such as respiratory depression. These results confirmed that dezocine did not increase dizziness and drowsiness, truncal rigidity, cold feeling, respiratory inhibition, nausea and emesis but was associated with a higher rate of dizziness, consistent with other meta-analyses.^[6,17]^

The present study had some limitations. First, due to the small sample size, the effects of confounding factors such as age, gender, smoking status, special comorbidities, and type of surgery on SIC were not further analyzed and discussed. Second, this study did not collect pain intensity indicators during anesthesia induction, and whether dezocine preconditioning interfered with opioid analgesia needed further research.

## 5. Conclusion

The current study suggested that premedication of 0.1 mg/kg dezocine could be a clinically effective method for attenuating SIC during anesthesia induction in CABG surgery, but 0.05 mg/kg dezocine was not.

## Acknowledgments

The authors thank the colleagues and statisticians at Fuwai Hospital and Fuwai Yunnan Hospital for their indispensable help with data collection and analysis.

## Author contributions

**Conceptualization:** Chun-mei Xie, Yun-tai Yao.

**Data curation:** Chun-mei Xie, Li-xian He, Meng-qi Shen.

**Formal analysis:** Chun-mei Xie, Li-xian He, Meng-qi Shen, Yun-tai Yao.

**Funding acquisition:** Chun-mei Xie, Yun-tai Yao.

**Investigation:** Chun-mei Xie, Li-xian He, Meng-qi Shen, Yun-tai Yao.

**Methodology:** Chun-mei Xie, Li-xian He, Meng-qi Shen, Yun-tai Yao.

**Software:** Chun-mei Xie, Li-xian He, Yun-tai Yao.

**Writing – original draft:** Chun-mei Xie.

**Writing – review & editing:** Chun-mei Xie, Li-xian He, Meng-qi Shen, Yun-tai Yao.
